# Antioxidant and Antiproliferative Potentials of *Ficus glumosa* and Its Bioactive Polyphenol Metabolites

**DOI:** 10.3390/ph14030266

**Published:** 2021-03-15

**Authors:** Moses Mutuse Mutungi, Felix Wambua Muema, Festus Kimutai, Yong-Bing Xu, Hui Zhang, Gui-Lin Chen, Ming-Quan Guo

**Affiliations:** 1 CAS Key Laboratory of Plant Germplasm Enhancement and Specialty Agriculture, Wuhan Botanical Garden, Chinese Academy of Sciences, Wuhan 430074, China; mutungi.moses7@gmail.com (M.M.M.); fwambua83@mails.ucas.ac.cn (F.W.M.); festokim81@mails.ucas.ac.cn (F.K.); xuyongbing17@mails.ucas.ac.cn (Y.-B.X.); zhanghui183@mails.ucas.ac.cn (H.Z.); glchen@wbgcas.cn (G.-L.C.); 2 Sino-Africa Joint Research Center, Chinese Academy of Sciences, Wuhan 430074, China; 3 University of Chinese Academy of Sciences, Beijing 100049, China; 4 Innovation Academy for Drug Discovery and Development, Chinese Academy of Sciences, Shanghai 201203, China

**Keywords:** *Ficus glumosa*, polyphenols, HPLC-ESI-MS/MS, antiproliferative, antioxidant

## Abstract

*Ficus glumosa* Delile (Moraceae), a reputed plant that is used in herbal medicine, is of high medicinal and nutritional value in local communities primarily ascribed to its phytochemical profile. Currently, there are hardly any fine details on the chemical profiling and pharmacological evaluation of this species. In this study, the flavonoids and phenolics contents of the ethanol extracts and four extracted fractions (petroleum ether (PE), ethyl acetate (EA), *n*-butanol, and water) of the stem bark of *Ficus glumosa* were firstly quantified. Further, their antioxidant and antiproliferative potentials were also evaluated. The quantitative determination indicated that the EA and *n*-butanol fractions possessed the highest total flavonoids/phenolics levels of 274.05 ± 0.68 mg RE/g and 78.87 ± 0.97 mg GAE/g, respectively. Similarly, for the 2,2-diphenyl-1-picrylhydrazyl (DPPH), 2,2′-azino-bis-(3-ethylbenzothiazoline-6-sulfonic acid) (ABTS), and ferric-reducing antioxidant power (FRAP) assays, the EA fraction exhibited high potency in both DPPH and ABTS^+^ scavenging activities with IC_50_ values of 0.23 ± 0.03 mg/mL, 0.22 ± 0.03 mg/mL, and FRAP potential of 2.81 ± 0.01 mg Fe^2+^/g, respectively. Furthermore, the EA fraction displayed high cytotoxicity against human lung (A549) and colon (HT-29) cancer cells. Additionally, the liquid chromatography coupled with electrospray ionization tandem mass spectrometry (LC-ESI-MS/MS) was employed in order to characterize the chemical constituents of the EA fraction of *Ficus glumosa* stem bark. Our findings revealed 16 compounds from the EA fraction that were possibly responsible for the strong antioxidant and anti-proliferative properties. This study provides edge-cutting background information on the exploitation of *Ficus glumosa* as a potential natural antioxidant and anti-cancer remedy.

## 1. Introduction

*Ficus glumosa* (Moraceae), which is also referred to as ‘African rock fig’, is a medium-sized tree, indigenous in semi-tropical and tropical African countries, and parts of West Asia [1]. It is a reputed species in African Traditional Medicine (ATM) for millennia and it serves a multipurpose use as food, healing diseases/ailments, and dye production. For instance, the leaves are boiled or eaten raw as vegetables in northern Nigeria [2], whilst the latex and figs as folk remedies, and the bark is used in the production of dye in the Southern, Eastern, and Western African countries [3]. Interestingly, the “swelling” trait of the latex makes it ideal as a potential disintegrant in tablet formulations [4]. Traditionally, figs and bark decoctions were used for the curing of rheumatoid arthritis, diarrhea, hemorrhoids, females sterility, and gingivitis [5,6], water retention, constipation, and liver complications [7]. The decoction from the leaves was orally administered for enhancement of weight loss, alleviate coughing, and helminths removal [8]. In Kenya, local communities use it for therapeutics and alimentation, for example, the figs are eaten as wild fruits and the latex is used to treat cancer complications and epilepsy [9].

Presently, some unraveled pharmacological activities of *Ficus glumosa* mainly include antidiabetic [10], antihypertension [11], hypolipidemia and antiatherogenic [12], antimalarial [13], antirheumatic [14], antioxidant [15], antibacterial [16], antifungal, and anticancer [17]. Further, studies on other *Ficus* species show substantial antiproliferative, anti-inflammatory, and antioxidant properties [18,19,20]. Previous studies linked the antioxidant, antibacterial, antitumor, and hypoglycemic properties of *Ficus glumosa* to its secondary metabolites profile, which is composed of alkaloids, flavonoids, saponins, triterpenoids, tannins, phenolic acids, steroids, and coumarins [21,22]. These polyphenols are naturally synthesized in plants as natural antioxidants and anticancer metabolites. They regulate cellular mechanisms that elicit antiproliferative activities. Further, they modulate the cumulative deleterious effects that are consequent of Reactive Oxygen and Nitrogen species (ROS/RNS) overproduction, growth factor receptors interaction, and pathways of cell signaling [23].

Antioxidants neutralize free radicals to suppress their oxidation potential by targeting signaling pathways, including phosphatidylinositide 3-kinases/protein kinase B (PI3K/Akt), among others [24]. Currently, there is an increased preference for natural antioxidants, as most synthetic antioxidants suffer a major setback of being carcinogenic [25]. The antioxidant and antiproliferative potentials of *Ficus glumosa* have been less explored, with a poor scientific elucidation of its medical utility. For instance, Ibrahim et al. [17] assayed the leaves of four species for antitumor properties (*F. glumosa*, *Holoptelea integrifolia*, *Ulmus parvifolia*, and *Rumex dantatus*), Madubunyi et al. [10] evaluated the antioxidant and antidiabetic potential of stem bark of *F. glumosa* in Nigeria, while Nana et al. [26] isolated chemical components from *F. glumosa* species and then assayed for their anticancer activities. Moreover, Olaokun et al. [27] screened for secondary metabolites, antioxidant properties, and inhibition of α-glucosidase and α-amylase enzymes of *Ficus glumosa*. The characterization of this species in Kenya is yet to be reported, despite having established that *Ficus glumosa* is rich in flavonoids, phenolic acids, fatty acids, and triterpenoids.

To this end, this study focused on evaluating the antioxidant and antiproliferative activities of ethanol extracts, ethyl acetate (EA), petroleum ether (PE), *n*-butanol, and water fractions of *Ficus glumosa*, and characterize its phytochemical profile. Meanwhile, the quantification of the total yield of flavonoids and phenolics of the ethanol extracts, PE, EA, *n*-butanol, and water fractions were performed. The high-performance liquid chromatography-electrospray ionization tandem mass spectrometry (HPLC-ESI-MS/MS) was further employed to identify the potential bioactive constituents in the EA fraction. Thus, this study will expand the scope of natural compounds screening and characterization, which serve as new entities in natural drug formulations.

## 2. Results and Discussion

### 2.1. Total Phenolics and Flavonoids Total Contents

Polyphenolics are ubiquitously synthesized in plants for growth and protection against invasion by predators and pathogens. They are similarly believed to have related effects on humans. For this reason, an evaluation of phenolics and flavonoids yield in this species was deemed to be necessary.

**Figure 1:**
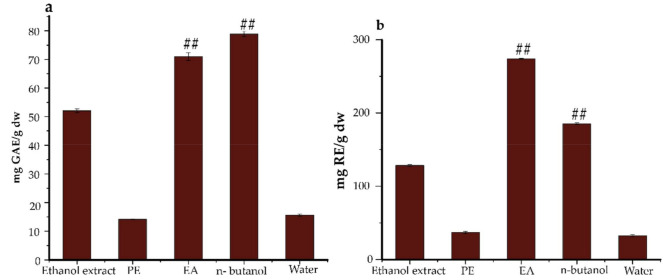
(**a**) (TPC) The total phenolics content and (**b**) (TFC) total flavonoids content of *Ficus glumosa*. The results are expressed in GAE (gallic acid equivalents) and RE (rutin equivalents) based on dry weight samples. ^##^ *p* < 0.01 as compared to ethanol extracts. PE, Petroleum ether; EA, ethyl acetate.

The total flavonoids and phenolics compositions (TFC & TPC) in *Ficus glumosa* ethanol extracts and fractions were determined while using two equations that were obtained from standard calibration curves: y = 1.535x − 0.0335, R^2^ = 0.9982 for TFC, and y = 1.9908x − 0.0202, R^2^ = 0.9993 for TPC. For TFC (Figure 1a), the EA fraction exhibited the highest content of 274.05 ± 0.68 mg RE/g, followed by *n*-butanol fraction with 185.34 ± 1.30 mg RE/g. Moreover, for TPC (Figure 1b), the *n*-butanol fraction showed the highest phenols accumulation of 78.87 ± 0.97 mg GAE/g, followed by the EA fraction with 70.99 ± 1.40 mg GAE/g. The observed trends of both TFC and TPC were in descending order of EA > *n*-butanol > ethanol extracts > PE > water, (8.44:5.71:3.96:1.14:1) and *n*-butanol > EA > ethanol extracts > water > PE, (5.56:5.01:3.67:1.10:1), respectively.

The present TPC yield in our study is lower when compared with that of methanol stem bark extract of *Ficus racemosa* that was obtained from Bangladesh, which was 242.97 mg GAE/g [28]. The TPC in *Ficus racemose* extracts from India was relatively lower, 39.03 ± 0.92 mg GAE/g extract [29]. Similarly, Abdel-Hameed [30] in a previous study on *Ficus decora,* and *Ficus afzelli* quantified TPC of 60.40 ± 3.06 and 70.96 ± 4.64 mg GAE/g in *n*-butanol fractions, 63.61± 3.70 and 97.30 ± 7.14 mg GAE/g in EA fractions, respectively. Further, the TFC levels of EA and *n*-butanol fractions of *Ficus glumosa* in the present study were higher than those of the same fractions of two *Ficus* species, *Ficus lyrata* and *Ficus sycomorous*, which were obtained from Egypt and Oman. They had TFC yields of 68.27 ± 4.17, 89.12 ± 6.88 mg RE/g, 153.52, and 123.54 mg QE/100g, respectively [30,31]. The extraction methodology and features of extracting solvents affect the solubility of different chemical components [32]. The TFC/TPC slight yields variation witnessed might be a consequence of the aforementioned factors, in addition to the difference in extraction procedures and geographical location.

### 2.2. Antioxidant Potential

**Figure 2:**
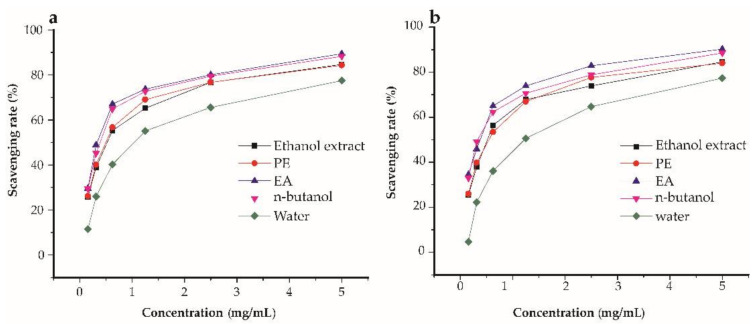
The percentage (%) scavenging rates of ethanol extracts, petroleum ether (PE), ethyl acetate (EA), *n*-butanol, and water fractions of *Ficus glumosa*. (**a**) The % radical scavenging rates of DPPH (2,2-diphenyl-1-picrylhydrazyl) assay, and (**b**) ABTS (2,2′-azino-bis (3-ethylbenzothiazoline-6-sulfonic acid) assay exhibited by ethanol extracts, PE, EA, *n*-butanol, and water fractions of *Ficus glumosa*.

**Figure 3:**
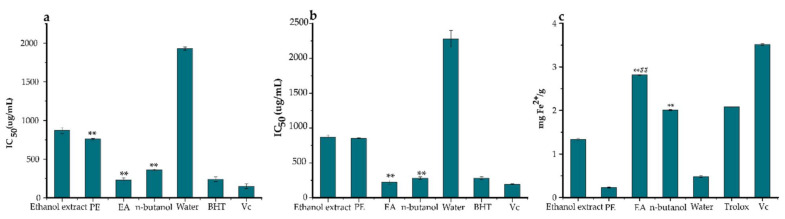
The antioxidant activities of ethanol extracts, petroleum ether (PE), ethyl acetate (EA), *n*-butanol, and water fractions of *Ficus glumosa* in IC_50_ and mg Fe^2+^/g. (**a**) The IC_50_ values of DPPH (2,2-diphenyl-1-picrylhydrazyl) assay, (**b**) ABTS (2,2’-azino-bis (3-ethylbenzothiazoline-6-sulfonic acid) assay, and (**c**) FRAP (ferric-ion reducing power) assay exhibited by ethanol extracts, PE, EA, *n*-butanol, and water fractions of *Ficus glumosa*, and positive controls; BHT (butylated hydroxytoluene), Vc (vitamin c). ** *p* < 0.05 and ^##^ *p* < 0.05 as compared to ethanol extracts and Trolox, respectively.

Evaluating the antioxidant potential of compounds using only one assay is considered to be inconclusive due to the complex nature of polyphenols and divergent mechanisms of free radical scavenging [33]. Three antioxidant assays were used in this study, namely DPPH, ABTS, and FRAP were conducted to evaluate and compare the antioxidant power of the ethanol extracts, PE, EA, *n*-butanol, and water fractions of *Ficus glumosa*. A general observation was that the ethanol extracts, PE, EA, *n*-butanol, and water fractions expressed certain scavenging potentials against DPPH and ABTS assays (Figure 2a,b). The DPPH assay (Figure 3a) showed that the EA fraction exhibited the highest potential with an IC_50_ value of 0.23 ± 0.03 mg/mL. Similarly, in the ABTS assay (Figure 3b), the EA fraction presented significant scavenging activity with a lower IC_50_ value of 0.22 ± 0.03 mg/mL than the control, BHT, which had an IC_50_ value equal to 0.28 ± 0.02 mg/mL. Meanwhile, in the FRAP assay (Figure 3c), the EA fraction also displayed the strongest antioxidant potential with the IC_50_ value of 2.81 ± 0.01 mg Fe^2+^/g, followed by *n*-butanol fraction and ethanol extracts. Generally, the EA fraction emerged as the most potent antioxidant in the three bioassays.

In previous studies on barks of four *Ficus* species, namely *F. glumosa* from Swaziland, *F. racemose* from Bangladesh, *F. racemose* from India, and *F. platyphylla* from Burkina Faso, displayed relatively lower DPPH IC_50_ values of 5.84 ± 1.53, 19, 5.99, and 1.93 ± 0.11 µg/mL, respectively [13,28,34,35]. Partly, our antioxidant results for DPPH and ABTS bear a close resemblance to those that were displayed by *Ficus* species from Yunnan, China [36]. They showed DPPH IC_50_ values, as follows; *Ficus virens var. verins* (1.03 mg/mL), *F. virens var. sublanceolata* (0.34 mg/mL), *F. callosa* (0.95 mg/mL), *F. oligodon* (2.54 mg/mL), *F. racemose* (1.11 mg/mL), *F. vasculosa* (0.97 mg/mL), and *F. auriculata* (0.29 mg/mL). Moreover, the same *Ficus* species exhibited ABTS IC_50_ values of 0.48, 0.23, 0.35, 0.86, 0.42, 0.69, and 0.25 mg/mL, respectively. Furthermore, our FRAP findings were consistent with previous studies that were reported by Madubunyi et al. [10] on *Ficus glumosa* bark extracts from Nigeria. The antioxidant activity correlates to the active metabolites in this sample fraction. The strong ability of polyphenols to donate H-atom to free radicals makes them suitable natural antioxidants [37]. In this regard, gallic acid, catechin, cinchonain I, cinchonain II, and procyanidin B2 are some well-known compounds with antioxidant potential that have been characterized in this study, hence being partly linked to the antioxidant results.

### 2.3. Antiproliferative Activity

**Figure 4:**
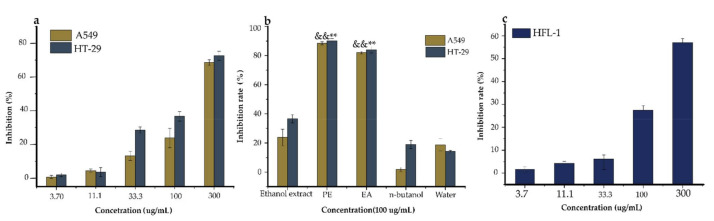
The antiproliferative activity of the ethanol extracts, PE (petroleum ether), EA (ethyl acetate), *n*-butanol, and water fractions of *Ficus glumosa*. (**a**) The (%) inhibition rates of HT-29 and A549 cells by different concentrations of ethanol extracts of *Ficus glumosa.* (**b**) The % inhibition rates of A549 and HT-29 cells treated with ethanol extracts, PE, EA, *n*-butanol, and water fractions of *Ficus glumosa*. (**c**) The toxicity of ethanol extracts of *Ficus glumosa* on normal lung (HFL-1) cells. ^&&^ *p* < 0.01, ** *p* < 0.01 when compared to ethanol extracts.

Anticancer activity was related to antioxidant activity, because antioxidants can suppress the formation of cancers that arise from oxidative stress. The effective regulation of ROS via an antioxidant system could reduce cancer manifestation and also help in cancer treatment [38]. Upregulated ROS generation promotes oxidative stress, particularly on DNA molecules, by altering some gene sequences and/or activating proto-oncogenes, causing DNA damage and resulting in mutations [39]. Polyphenols elicit antiproliferative activities through the regulation of some transduction pathways, such as signal phosphatidylinositol 3-kinase (P13K), transducer and activating transcription (STAT-1), Nuclear factor-like 2 (erythroid-derived 2-) (Nrf2), Hypoxia-inducible factor (HIF)-1 Peroxisome proliferator-activated receptor (PRAR), and Mitogen-activated kinase-like protein (MAKP) [40]. Thus, it was important to assay the ethanol extracts, EA, *n*-butanol, PE, and water fractions of *Ficus glumosa* for antiproliferative activity on two cancer cells, namely lung carcinoma (A549) and colon carcinoma (HT-29) using SRB (sulforhodamine B) assay (Figure 4).

First of all, the cytotoxic effect of the ethanol extracts of *Ficus glumosa* was examined (for a concentration range from 3.7 to 300 µg/mL) on HT-29 and A549 cells over 72 h. It was revealed that the ethanol extracts exhibited dose-dependent inhibitory activities on HT-29 and A549 cells, with IC_50_ equal to 124.40 and 186.10 µg/mL, respectively. Aliquots of 100 µg/mL of ethanol extracts, PE, EA, *n*-butanol, and water fractions were evaluated for the antiproliferative activity. The PE and EA fractions displayed significant cytotoxicity, with an inhibition of 90.26% and 84.04% on HT-29 cells, and 88.38% and 82.10% on A549 cells, respectively, in comparison with the other fractions and ethanol extracts. The SRB assay of ethanol extracts on HFL-1 cells exhibited a high IC_50_ value of 232.66 µg/mL, which indicated low/no toxicity on a normal human lung. This indicated that the sample was biocompatible with the cell line. In regards to previous studies on stem barks of *Ficus* species (*F. fistulosa, F. hispida,* and *F. schwarzii*), they displayed relatively higher antiproliferative activities on A549 and HT-29 cells [41]. Similar findings were also reported in another study on *Ficus drupacea* stem bark extract against HT-29 cells, which unraveled an anticancer potential of IC_50_ 28. 9 ± 3.7 µg/mL [42]. Additionally, this study upholds the potential of this species to address a broad range of multifaceted diseases. Notably, the subject pharmacological potential of this species could be linked with the quality and quantity of structurally diverse secondary constituents’ interaction with the test components.

### 2.4. HPLC Method Validation

The developed procedure was initially validated as ready for the quantification of the phenolic derivatives, phenolic acids, and flavonoids characterized in the EA fraction of *Ficus glumosa.* The calibration curves and correlation coefficients for the two standards were higher than R^2^ > 0.998; an indication of good linearity within the examined ranges. Meanwhile, the LODs for both gallic acid and rutin were 1.84 and 1.93 µg/mL, whereas the LOQs were 5.58 and 5.85 µg/mL, respectively. This depicted a high sensitivity and reliability of the procedure for the effective quantification of analytes.

Further, our method was regarded to be repeatable, precise, and highly stable exhibited by the RSDs of intraday precision, repeatability, stability, and interday precision tests, which were all <2.5%. The recoveries ranged between 95–99% with RSDs of <2.43% indicating high accuracy and reproducibility. Table 1 provides the validation parameters results.

### 2.5. HPLC-MS Analysis of EA Fraction of Ficus glumosa

**Figure 5:**
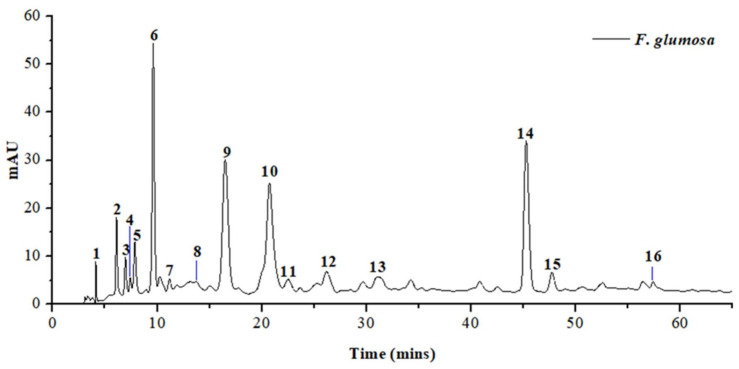
High-performance liquid chromatography-UV (HPLC-UV) chromatogram of EA fraction of *Ficus glumosa* at wavelength 280 nm.

**Figure 6:**
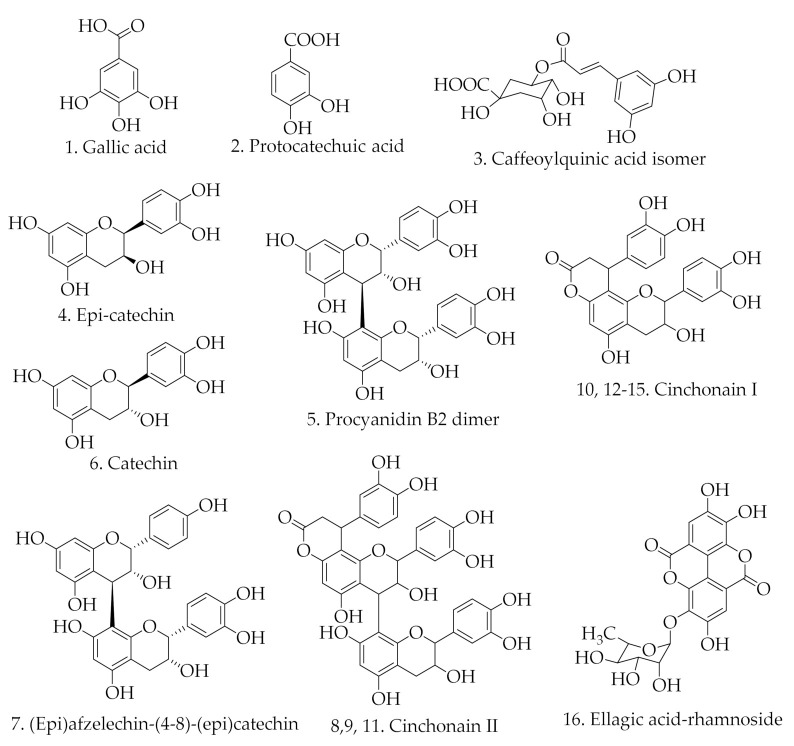
Structures of constituents tentatively identified in the EA fraction of *Ficus glumosa* stem bark.

In this study, for the first time, the qualitative analysis of the EA fraction of *Ficus glumosa* stem bark was achieved using HPLC-ESI-MS/MS (Figure 5). The HPLC method was first optimized to allow clear separation and maintain proper peak shapes in order to ensure a detailed investigation. An in-depth chromatographic investigation that was based upon retention time, the order of elution, and MS base peak led to the characterization of 16 compounds; three phenolic acids, a phenolic derivative, and 12 flavonoids, as in Table 2. The structures of the characterized compounds are shown in Figure 6. 

Compound **1** produced the [M-H]^−^ at *m/z* 169, and the characteristic fragment ions at *m/z* 169 and 125, which signified a neutral loss of CO_2_ molecule (44 Da). Thus, compound **1** was tentatively identified as gallic acid, as per recent reports [43,44]. Compound **2** displayed a precursor molecular ion [M-H]^−^ at *m/z* 153, and the loss of carboxyl group (44 Da) between the major fragment ions at *m/z* 153 and *m/z* 109 an indication of decarboxylation. The deprotonation of a molecule ion at *m/z* 153 led to the tentative identification of compound **2** as protocatechuic acid [45].

Compound **3** exhibited the [M-H]^−^ at *m/z* 353 and produced the fragment ion at *m/z* 191 [quinic acid-H]^−^ due to a loss of caffeoyl group (C_9_H_6_O_3_). Compound **3** was tentatively identified as caffeoylquinic acid isomer, as earlier reported in xiao-er-qing-jie (XEQJ) granules and coffee [46,47].

**Figure 7:**
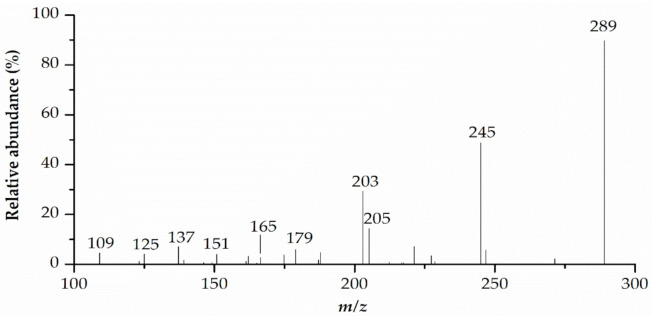
MS/MS spectra for catechin (peak **6**). The peak number corresponds to that in Table 2.

**Figure 8:**
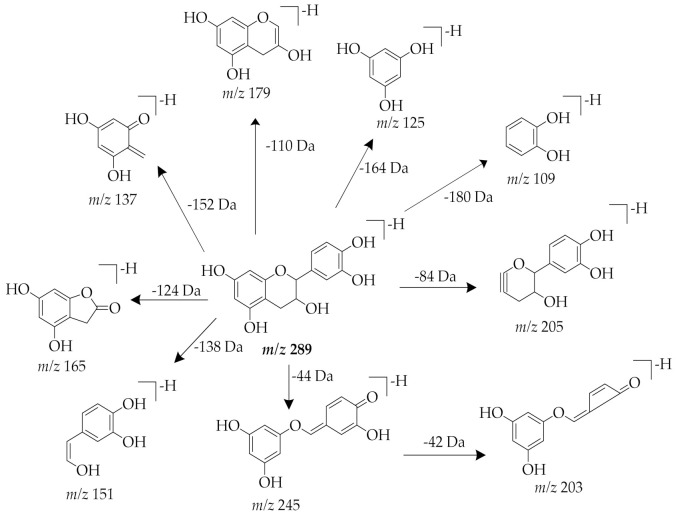
Proposed fragmentation pathway for catechin (peak 6).

Compounds **4** and **6** showed a similar protonated precursor ion at *m/z* 335 [M+HCOOH-H]^−^, which corresponds to formate adduct [M+HCOO]^−^ that formed from formic acid in the A-mobile phase. The MS^2^ ion at *m/z* 289 generated fragments at *m/z* 245 [M-H-C_2_H_4_O]^−^, 205 [M-H-84]^−^, 203 [M-H-C_2_H_4_O-C_2_H_2_O]^−^, 179 [M-H-110]^−^, 165 [M-H-124]^−^, 151 [M-H-C_7_H_6_O_3_]^−^, 137 [M-H-152]^−^, 125 [M-H-164]^−^, and 109 [M-H-180]^−^. The fragment ion at *m/z* 245 resulted from a loss of a C_2_H_4_O group. The cleavage of A ring produced the fragment ion at *m/z* 205. The elimination of a catechol group (C_6_H_6_O_2_) yielded the ion at *m/z* 179. After heterocyclic ring fission (HRF), the B-ring was eliminated, generating fragment ion at *m/z* 165. The loss of rings A and C yielded ion at *m/z* 109. The ion at *m/z* 203 was produced from a molecular ion at *m/z* 245 after cleavage at the C-ring. Analyzing the elution order, retentions, and abundance of the fragments, the two compounds were concluded as isomers. When comparing the MS^2^ spectra data and proposed fragmentation mechanism (Figure 7 and Figure 8) of these compounds with recent data reports, compound **4** and compound **6** were tentatively identified as epicatechin and catechin, respectively [48,49].

Compound **5** presented the [M-H]^−^ at *m/z* 577. Upon deprotonation, it yielded MS^2^ at *m/z* 425, 407, 289, 245, 161, 137, and 125, respectively. The ion at *m/z* 425 resulted from the RDA (Retro-Diels-Alder) reaction losing 152 Da, then water (H_2_O) was eliminated to form the molecular ion at *m/z* 407. The daughter ion at *m/z* 289 was consequent of the cleavage of quinone methide (QM) at the interflavan bond. The molecular ion at *m/z* 289 underwent further deprotonation to produce ions at *m/z* 245, 137, and 125, due to the neutral elimination of C_2_H_4_O molecule, RDA reaction, and a loss of 164 Da, respectively. The loss of 84 Da from ion at *m/z* 245 produced ion at m/z 161. When comparing the MS/MS fragmentation of this compound with bibliographic data [50], compound **5** was tentatively identified as procyanidin B2 dimer.

Compound **7** showed the [M-H]^−^ at *m/z* 561. After the RDA reaction that was characterized by a loss of 152 Da, it yielded ion at *m/z* 437. Further, MS^2^ at *m/z* 289, 273, and 271 were generated as a result of quinone methide cleavage at interflavan bond. The molecular ion at *m/z* 289 suffered further deprotonation and released molecule ion at *m/z* 245 after losing a C_2_H_4_O group (44 Da). By analyzing this fragmentation mechanism, compound **7** was tentatively identified as (Epi)afzelechin-(4-8)-(epi)catechin, which was reported earlier in *Laurus nobilis* wood [51].

Compounds **8**, **9**, and **11** shared [M-H]^−^ ions at *m/z* 739 with common MS/MS spectra at *m/z* 569, 459, 435, 417, 289, and 177, implying that they are isomers. The ion at *m/z* 569 [M-H-152-H_2_O]^−^ was produced as a consequence of RDA cleavage at the C ring characterized by 152 Da loss, and then followed by H_2_O molecule loss. The fragment ion at *m/z* 459 [M-H-152-H_2_O-C_6_H_6_O_2_]^−^ was generated due to the loss of dihydroxybenzene (C_6_H_6_O_2_) from ion at *m/z* 569. Quinone methide fission generated ion at *m/z* 289, which also produced the daughter ion at *m/z* 177 after a loss of 112 Da. Repeated RDA cleavage at C-rings of lower and upper subunits led to the formation of the molecular ion at *m/z* 435. The ion at *m/z* 435 underwent a loss of H_2_O molecule to form the fragment ion at *m/z* 417. Hence, compounds 8, 9, and 11 were concluded as cinchonain II isomers. To our expectation, similar compounds with identical fragmentation were reported in *Crataegus folium* and *Inula viscosa* species, respectively [52,53].

Compounds **10**, **12**, **13**, **14**, and **15** displayed the [M-H]^−^ at *m/z* 451. They produced characteristic MS^2^ fragments at *m/z* 341 [M-H-C_6_H_6_O_2_]^−^, *m/z* 231 [M-H-2C_6_H_6_O_2_]^−^, *m/z* 217 [M-H-2C_6_H_6_O_2_-CH_2_]^−^, and *m/z* 189 [M-H-2C_6_H_6_O_2_-C_2_H_2_O]^−^, respectively, a confirmation that they were isomers. Besides, fragment ion at *m/z* 217 underwent further loss of 40 Da to generate predominant ion at *m/z* 177. Compounds **10**, **12**, **13**, **14**, and **15** were identified as cinchonain I isomers, which were earlier reported in *Acer palmatum* by Zhang et al. [54].

Compound **16** exhibited [M-H]^−^ at *m/z* 447, and then released a series of MS^2^ ions at *m/z* 403 [M-H-44]^−^, 323 [M-H-124]^−^, and 295 [M-H-124-CO]^−^. By comparing this fragmentation mechanism with MS/MS data in previous literature, compound **16** was tentatively identified as ellagic acid-rhamnoside [55].

### 2.6. Quantification of Polyphenols in EA Fraction of Ficus glumosa

The biological assays and therapeutic effects of this species depend on the quantity of each secondary metabolite identified. Particularly, the efficacy of traditional drugs relies on the phytochemicals traits that elicit different biological activities.

The validated methods mentioned above were employed to investigate the quantities of the 16 bioactive constituents that were identified in the EA fraction of *Ficus glumosa*, as per the previous study. The secondary metabolites that were characterized in this fraction were polyphenols, known for their pharmacological properties. Each polyphenol was quantified based on two linear curves that were developed using external standards (gallic acid and rutin) and plotting the areas of established curves against concentrations. The phenolic derivatives and phenolic acids were given in terms of gallic acid, whereas the flavonoids were expressed as rutin. The quantities of the identified compounds in the EA fraction of *Ficus glumosa* stem bark were estimated and Table 2 provides their quantification results. As confirmed in the screening data, the flavonoids were mainly flavanols and they were the most chemical constituents in *Ficus glumosa* in this study. Cinchonain I isomer 3 (14) was the most abundant constituent, with 31.76 µg/g. Cinchonain II isomer 1 (9) was the second in abundance, with 30.86 µg/g, followed by catechin (6) (22.93 µg/g). Cinchonain I isomer 2 (13) was the least abundant, with 0.29 µg/g. Generally, cinchonain I isomers accounted for higher content, followed by cinchonain II isomers in this study. Phenolic acids occurred in trace amounts. In this class of compounds, protocatechuic acid displayed a higher content of 3.83 µg/g when compared to the other phenolic acids. Hence, it is suspected flavonoids accounted for the promising biological activities depicted by this fraction, owing to their abundance. It is worth noting that the quantification of each compound content was an estimation of occurrence in the analyzed sample.

The chemical and content variation in this species might affect further pharmacological explorations of *Ficus glumosa*. The geographical location and period of samples’ collection might have influenced the content of bioactive constituents in this species. In addition to the aforementioned factors, perhaps there might be other factors affecting the chemical content that require extra investigations. Further, enhancing the efficacy, precision, and consistency in the preparation of herbal drugs, the validation of every step involved is recommended.

### 2.7. Biological Significance of Chemical Constituents in EA Fraction of Ficus glumosa

The pharmacological activities of this species could be linked with the quantity and quality of structurally diverse secondary constituents that interact and react with the test components. HPLC-ESI-MS/MS analysis of EA fraction revealed 16 polyphenolic compounds, mainly flavonoids and phenolic acids (Table 2). The aforementioned compounds are well known for their bioactivity significance.

Polyphenols are normally formed in plants for obvious roles; however, their increased synthesis is triggered as a counter mechanism to biotic/abiotic stresses [56]. Some of the identified compounds belong to phenolic acids, namely gallic acid (1), protocatechuic acid (2), and caffeoylquinic acid isomer (3). These are active metabolites that are widely reported with strong antioxidant and antitumor bioactivities [57,58]. The number of OH groups, the saturation degree, and other substitute groups dictate their potential in radical quenching/scavenging mechanism as well as their anticancer activity [59].

Flavonoids possess versatile health-promoting effects, as demonstrated in previous studies [60]. Majorly, flavanols constituted a higher percentage of components in the EA fraction. Bansal [61] highlighted that flavanols’ biological properties tend to be influenced by the position and number of OH groups, the presence of catechol/pyrogallol groups on the B-ring, OH groups at positions C3, C5, and C7, and their degree of polymerization. In this regard, bioactivity decrease from trimers to monomers, whilst it increases from trimers to tetramers, whereas the ease of degradation varies with the type of interflavan bond in oligomers, with epicatechin being more easily oxidized than catechin [62]. In the present study, epicatechin (4), procyanidin B2 dimer (5), catechin (6), (epi)afzelechin-(4-8)-(epi)catechin (7), cinchonain II (8), and cinchonain I (10) were tentatively identified in this species and they were previously reported with some biological importance. They possess antioxidant, anti-inflammatory, and antiproliferative properties for the case of catechin (6) and epicatechin (4) [63], procyanidin B2 dimer (5) [64], and (epi)afzelechin-(4-8)-(epi)catechin (7) [65]. Further, cinchonain I (10) and II (8) compounds are reported to depict good antioxidant potential [66,67]. Ellagic acid-rhamnoside (16) was earlier assayed and found to exhibit high antioxidant and antibacterial properties [68,69].

Each of the identified compounds is believed to have interacted with the test components at different affinities that are reflected in the biological activities. Moreover, these identified phytochemicals showed lower potency than the standard controls that were used in the experimental tests. Hence, we recommend thorough screening and elucidation, followed by biological activity evaluation of each compound.

## 3. Materials and Methods

### 3.1. Chemicals and Reagents

China Medicine (Group) Shanghai Chemical Reagent Corp provided ethanol, ethyl acetate, petroleum ether, hexane, and *n*-butanol. (Shanghai, China). Acetonitrile and formic acid (HPLC grade solvents) were acquired from TEDIA Limited (Fairfield, OH, USA) and used with no further purification. The ultrapure water for LC-MS/HPLC analysis was generated using an EPED machine (Yeap Esselte Tech. Co., Nanjing, China). Sulforhodamine B (SRB), Dulbecco’s Modified Eagle Medium (DMEM), streptomycin, and dimethyl sulfoxide (DMSO) were purchased from Gibco (New York, NY, USA). Millipore membranes (0.22 μm) were provided by Jinteng Instrument Corporation (China). Trolox, gallic acid (99%), rutin (98%), vitamin C, BHT, ABTS, DPPH, and TPTZ were bought from Sigma–Aldrich Corporation (St. Louis, MO, USA).

### 3.2. Plant Materials

The fresh stem barks of *Ficus glumosa* were harvested from a farm in Makueni County, Kenya in July 2019. The cultivated plant materials were identified and authenticated by a botanist from the East Africa Herbarium, National Museums of Kenya. A voucher specimen number EAHF001/2019 was deposited in the herbarium of East Africa.

These materials were washed, dried under a shed, pulverized, powdered, and then packed into polythene bags. They were stored at room temperature for further analysis.

### 3.3. Sample Extraction and Partitioning

The extraction process was performed as per a previous study [70] with some modifications. The 4.0 kg dried powdered *Ficus glumosa* stem bark was repeatedly extracted (30 min. for three times) using an ultrasound-assisted extraction method at 30 °C with 70% ethanol. The extracts were then combined and filtered. A rotatory evaporator was used to evaporate the filtered extracts at 45 °C under reduced pressure and then lyophilized for 48 h. The total dry ethanol extracts obtained was 435.5 g. From the dry ethanol extracts, 39.5 g was dissolved into 200 mL of H_2_O. This solution was then subjected to subsequent liquid/liquid extraction using petroleum ether (PE), ethyl acetate (EA), *n*-butanol, and water in that order to obtain their corresponding PE, EA, *n*-butanol, and water fractions.

### 3.4. Preparation of Standard Solutions

Two reference compounds, rutin, and gallic acid, were prepared while using methanol, each at 1 mg/mL. The standard solutions were properly attenuated using methanol in a serial dilution to obtain working standards. The working solutions/standards ranged from 1.25 to 40 µg/mL for rutin and 1 to 32 µg/mL for gallic acid. Each of the external calibration curves was established using six working standards.

### 3.5. Method Validation for Quantitative Analysis

This analysis was conducted as per a recent study [71] with some modifications. The established procedure was used in determining the limit of detection and quantification (LOD/LOQ), precision, stability, and accuracy. Firstly, 10 µL of each of the working standards was analyzed using HPLC-UV (Agilent 1220 HPLC, Waldron, Germany) and chromatograms were obtained at 280 nm. Peak areas were plotted versus concentrations to obtain linear graphs. The LODs and LOQs were examined at a signal-to-noise ratio (S/N) of 3.3 and 10, respectively.

Intraday precision tests were conducted by analyzing one sample in six replicates within one day. The same sample was analyzed for three consecutively days in triplicate to evaluate for interday precision. For the repeatability test, five replicates of the sample extract were analyzed in a day. The stability test was conducted by examining one sample six times.

Two lots of three replicates of the sample extract were prepared to investigate for recovery. The first lot was analyzed. The second lot was spiked with a known concentration of the standards and then analyzed. Recovery (%) was calculated, as follows: [Detected amount—initial amount]/spiked amount × 100%. Eventually, precision, repeatability, stability, and recovery were given in RSD %.

### 3.6. Determination of Total Flavonoid Content (TFC)

This analysis was determined colorimetrically, as per [72] with few adjustments. Firstly, a freshly prepared sample was mixed well with 0.2 mL of 5% NaNO_2_ in a 4 mL EP tube for 6 min. Afterward, 0.2 mL of 10% AlCl_3_·6H_2_O was added and the solution was allowed to settle. After 6 min., 1 mL of 4% NaOH solution was added. The contents were left for 15 min., after which the absorbance was observed at 510 nm with a UV-spectrophotometer (UV-11000, MAPADA Shanghai, China). This procedure was repeated using rutin in place of a sample extract. This test was performed in triplicate and the results were defined as mg of rutin equivalents per gram (mg RE/g) sample.

### 3.7. Determination of Total Phenolic Content (TPC)

The TPC was performed in accordance with the Folin–Ciocalteu guidelines [73], with some modifications. First, 50 µL of prepared sample extract together with 1.0 mL of 2% Na_2_CO_3_ solution were added in a 2.0 mL EP mixed, and then incubated for 6 min. After 50 µL Folin–Ciocalteu reagent addition, the solution was incubated in darkness for 40 min. Finally, the reaction absorbance was observed at 750 nm with a UV-spectrophotometer (UV-1100, MAPADA Shanghai, China). This method was repeated by replacing the sample extract with gallic acid. For each sample, this assay was conducted in triplicate, and the results were given as mg of gallic acid equivalents (mg GAE/g) per gram of sample.

### 3.8. In-Vitro Antioxidant Assays of Ficus Glumosa

#### 3.8.1. DPPH Assay

The DPPH assay of the Ficus glumosa samples was evaluated as per the previous method [74] with little modifications. DPPH solution (0.1mM) was prepared using methanol. First, 10 µL of the prepared sample extract and positive controls were mixed each interchangeably with 190 µL of already prepared DPPH in a well-plate. The mixture was cultivated for 30 min. in the dark and the attained absorbance was observed at 517 nm using the multifunctional 96-well plate reader (Tecan, Infinite M20PRO, Switzerland). Methanol was used to correct the baseline as the blank. This assay was repeated three times. The percentage rate of the DPPH scavenging was calculated and then expressed, as follows: DPPH activity (%) = [(C_O_-C_1_/C_O_)] × 100%, where Co is the control absorbance and C_1_ is the sample extract/control absorbance.

#### 3.8.2. ABTS Assay

This assay was performed as per [75] with minimal modifications. The ABTS solution (7 mM) was first prepared using ultrapure water. After that, ABTS^+^ stock solution preparation was done by mixing equivalent volumes of ABTS (7 mM) with (2.45 mM) potassium persulfate (dissolved in water), and then the mixture was incubated for 16 h. Methanol was used to dilute the ABTS^+^ stock until an absorbance of 0.70 ± 0.02 was achieved at 734 nm. The test samples were then appropriately attenuated to attain serial concentrations. Subsequently, 10 µL of the freshly prepared sample was mixed with 190 µL ABTS^+^ solution and left covered for 30 min. The contents absorbance was observed and noted at 734 nm. Methanol was used to correct the baseline. This test was done three times. The ABTS calculation and results expression was in terms of IC_50_ similarly as in the DPPH assay that is described above.

#### 3.8.3. FRAP Assay

The FRAP assay test of ethanol extracts, petroleum ether (PE), ethyl acetate (EA), n-butanol, and water fractions of Ficus glumosa was done as per [70] with minimal modifications. First, the stock solution, FRAP reagent (Fe^3+^-TPTZ), was made up of 300 mM acetate buffer of pH 3.6 (sodium acetate, acetic acid plus water), FeCl_3_·6H_2_O solution, and 10 mM TPTZ (2,4,6-tri(2-pyridyl)-S-triazine) solution in 40 mM HCl in a ratio of 10:1:1 (*v*/*v*/*v*). It was heated first to attain 37 °C. In a 1.5 mL EP tube, 20 µL of the properly mixed sample was mixed with 60 µL H_2_O and 520 µL fresh FRAP reagent. This solution was stored for 12 min. at 37 °C. Afterward, the absorbance of the mixture was read at 593 nm with a UV-spectrophotometer (UV-1100, MAPADA, China). Each test was repeated three times. The results were eventually given as milligrams Fe^2+^ per gram (mg Fe^2+^/g) of the sample in mean values ± SEM. FeSO_4_·7H_2_O was used as the standard.

### 3.9. In-Vitro Anti-Proliferative Assay of Ficus glumosa

#### 3.9.1. Cell Culture

Two human cancer cells for colon adenocarcinoma (HT-29) and lung adenocarcinoma (A549) were both purchased from China Centre for Type Culture Collection (Hubei, China). The DMEM (checked for contamination prior use) for the culture of cancer cells was composed of 100 µg/mL streptomycin-1% penicillin (100 U/mL), fetal bovine serum (10%), and L-glutamine (2 mM). The cells were cultured in an environment of 90% relative humidity (RH), 5% CO_2_, and 37 °C for one week to achieve confluence. A hemocytometer was employed in order to check for the viable cells well suited for cytotoxicity assay with only those having over 80% confluence being the ones selected.

#### 3.9.2. Anti-Proliferative Activity Analysis

The ethanol extracts, petroleum ether (PE), ethyl acetate (EA), n-butanol, and water fractions of *Ficus glumosa* were evaluated for antitumor activity against A549 and HT-29 cancer cells using the sulforhodamine (SRB) assay [76] with minimal modifications. Briefly, 100 µL monolayer cells (of specified density) that were contained in DMEM were inoculated in each 96-well-plate. The cells were incubated for 3 h in a well-humidified area at 90% RH, 37 °C, and then 5% CO_2_ for another 24 h to regain confluence before the sample addition. At first, the extract samples were dissolved at high concentrations using DMSO, and later at a lower concentration using water and then allowed to dry at room temperature. DMEM was used to prepared different concentration samples in each well. The 96 well-plate was incubated for 72 h, and 5 µL of 10% cold (4 °C) trichloroacetic acid (TCA) was then added for 40 min. at 4 °C. The supernatant was washed and dried at 25 °C. Subsequently, the plates (dried) were stained in 1% CH_3_CO_2_H using 50 µL of 0.4% *w*/*v* SRB for 20 min. The 1% CH_3_CO_2_H was used to wash the plates and then allowed to dry. After drying, they were dissolved using 150 µL of 10 mM Tris base. The absorbance was taken at 510 nm wavelength. A medium with less than 0.1% DMSO was used as the control. This assay was conducted in triplicate and the results evaluated, as follows.

IC_50_ cell inhibition (%) = [OD mean of control − OD mean of extract/OD mean of control] × 100%, where OD is the absorbance value.

### 3.10. HPLC-ESI-MS/MS Analysis of EA Fraction of Ficus Glumosa

The analysis of *Ficus glumosa* was conducted using a Thermo Accela HPLC 600 connected with a mass spectrometer, TSQ-Quantum^TM^ Access MAX (Thermo Fischer, San Jose, California, USA). The separation of the sample was achieved by Waters Symmetry RP-C18 column, 4.6 × 250 mm, 5 µm (Milford, USA) at 30 °C. The solvents (water/acetonitrile) were composed of formic (FA) acid (0.1%) in ultrapure water (A mobile phase) and 100% ACN (B mobile phase). The HPLC elution gradient was adjusted and set, as follows: 15–17% in 0–5 min., 17% in 5–15 min., 17–23% in 15–40 min., 23–25% in 40–45 min., and 25–33% in 45–65 min. Injected volume, 10 µL, 0.8 mL/min. as the flow rate, and online monitoring of UV-chromatogram was at 280 nm. The MS settings were adjusted, as below: negative full scan and dependent-data scan mode, capillary temperature, 350 °C, vaporizer temperature at 300 °C, sheath gas pressure (N_2_) at 40 psi, auxiliary pressure (N_2_) at 10 psi, and spray voltage at 3kV and mass range at 150–1500 m/z.

### 3.11. Statistical Analysis

All analyses were conducted, and their resulted values were given as mean ± standard deviations. The SPSS statistics 22 software (IBM Corporation, New York, USA) was used for data analysis. One way ANOVA with Duncan test was used for comparing multiple means and significance differences were considered at *p* < 0.01 and *p* < 0.05.

## 4. Conclusions

In this study, all of the bioassays conducted serve as evidence of the effects of *Ficus glumosa* on ABTS, DPPH, and FRAP, along with antiproliferative activities on A549 and HT-29 cells. This is the first study to report on the phytochemical profile, antioxidant, and antiproliferative properties of the EA fraction of *Ficus glumosa* stem bark. The EA fraction depicted good potency in both antitumor and antioxidant activities, which reflected the polyphenols composition in this fraction. Notably, HPLC-ESI-MS/MS analysis led to the characterization of 16 compounds, which consisted of three phenolic acids, a phenolic derivative, and 12 flavonoids. Hence, this study comprehensively substantiates *Ficus glumosa* as a prospective remedy for cancer phytomedicine development. Meanwhile, detailed phytochemical fingerprinting work on this underexploited species not only provide a baseline for further investigations, but is also considered to be paramount in support of its pharmacological value, as justified in ethnopharmacological use.

## Figures and Tables

**Table 1:** Method validation for two compounds investigated.

Property	Analytes			
	Gallic Acid		Rutin	
Calibration equation	y = 24.287x − 21.448		y = 16.298x + 3.3106	
Linear ranges (µg/mL)	1–32		1.25–40	
Correlation coefficient (R^2^)	0.9982		0.9988	
LOD	1.84		1.93	
LOQ	5.58		5.85	
Intraday precision (% RSD)	2.36		0.58	
Interday precision (% RSD)	1.69		0.47	
Repeatability (% RSD)	2.10		0.65	
Stability (% RSD)	2.49		0.56	
Recovery	Average recovery (%)	% RSD	Average recovery (%)	% RSD
	95.95	2.43	98.17	0.55

y = peak area; × = concentration of analytes (µg/mL); R^2^ = correlation coefficient; LOD/LOQ, limit of detection/quantification (S/N = 3.3/10); % RSD = percentage relative standard deviation.

**Table 2:** High-performance liquid chromatography-electrospray ionization tandem mass spectrometry (HPLC-ESI-MS/MS) data of compounds that were obtained from the EA fraction of *Ficus glumosa* stem bark.

Peak No.	Rt (min)	[M-H]^_^	MS/MS Fragments	Tentative Identification	Content (µg/g)	References
1	4.13	168.92	169, 125	Gallic acid	1.59	[43,44]
2	6.11	153.03	153, 109	Protocatechuic acid	3.83	[45]
3	6.96	353.08	191	Caffeoylquinic acid isomer	2.36	[46,47]
4	7.43	335.16	289, 245, 205, 203, 179, 151, 137, 125, 109	Epi-catechin	0.74	[48,49]
5	7.86	577.23	425, 407, 289, 245, 203, 161, 137, 125	Procyanidin B2 dimer	4.28	[50]
6	9.62	335.11	289, 245, 205, 203, 179, 165, 151, 137, 125, 109	Catechin	22.93	[48,49]
7	11.17	561.19	289, 273, 271, 245	(Epi)afzelechin-(4-8)-(epi)catechin	1.14	[51]
8	13.79	739.33	569, 459, 435, 417, 289, 177	Cinchonain II	0.32	[52,53]
9	16.50	739.38	739, 587, 569, 459, 435, 417, 339, 289, 245, 177	Cinchonain II isomer 1	30.86	[52,53]
10	20.75	451.16	341, 231, 217, 189, 177	Cinchonain I	22.82	[54]
11	22.50	739.29	569, 477, 459, 449, 435, 417, 339, 289, 177	Cichonain II isomer 2	2.24	[52,53]
12	26.18	451.15	341, 289, 231, 217, 189	Cinchonain I isomer 1	4.24	[54]
13	31.42	451.14	341, 231, 217, 189, 177	Cinchonain I isomer 2	0.29	[54]
14	45.29	451.13	341, 231, 217, 189, 177	Cinchonain I isomer 3	31.76	[54]
15	47.76	451.12	341, 217, 189, 177	Cinchonain I isomer 4	3.88	[54]
16	57.42	447.19	447, 403, 323, 295	Ellagic acid-rhamnoside	1.00	[55]

RT: retention time. Compounds were identified by comparing the mass spectra with literature data and available standards from databases (PubChem, ChemSpider, HDMB, and MassBank). The content of each compound is expressed as µg/g of dry weight.

## Data Availability

Data sharing not applicable.
